# Differences in Lymph Node Metastases Patterns Among Non-pancreatic Periampullary Cancers and Histologic Subtypes: An International Multicenter Retrospective Cohort Study and Systematic Review

**DOI:** 10.1245/s10434-024-15213-z

**Published:** 2024-04-11

**Authors:** Bas A. Uijterwijk, Daniël H. Lemmers, Giuseppe Kito Fusai, Alessandro Zerbi, Roberto Salvia, Ernesto Sparrelid, Steven White, Bergthor Björnsson, Vasileios K. Mavroeidis, Keith J. Roberts, Michele Mazzola, Santiago Sánchez Cabús, Zahir Soonawalla, Dimitris Korkolis, Mario Serradilla, Patrick Pessaux, Misha Luyer, Nicholas Mowbray, Benedetto Ielpo, Alessandro Mazzotta, Jorg Kleeff, Ugo Boggi, Miguel Angel Suarez Muñoz, Brian K. P. Goh, Elena Andreotti, Hanneke Wilmink, Michele Ghidini, Alberto Zaniboni, Caroline Verbeke, Volkan Adsay, Denise Bianchi, Marc G. Besselink, Mohammed Abu Hilal, For the International Study Group on Non-Pancreatic Periampullary Cancer (ISGACA), Gennaro Nappo, Poya Ghorbani, Giuseppe Malleo, Francesco Lancelotti, Niccolò Napoli, Stuart Robinson, Khalid Khalil, Alejandro Ramirez-Del Val, Matthew C. M. Mortimer, Bilal Al-Sarireh, Ye Xin Koh, Ricky Bhogal, Alejandro Serrablo, Brice Gayet, Karin Johansen, Mark Ramaekers, Alessandro Giani

**Affiliations:** 1 Department of Surgery, Fondazione Poliambulanza, Brescia, Italy; 2 Department of Surgery, Amsterdam UMC, Location University of Amsterdam, Amsterdam, The Netherlands; 3 Cancer Center Amsterdam, Amsterdam, The Netherlands; 4 Department of Surgery, Royal Free London NHS Foundation Trust, London, UK; 5 Department of Biomedical Sciences, Italy and Pancreatic Surgery, Humanitas University, IRCCS Humanitas Research Hospital, Pieve Emanuele, Rozzano, Italy; 6 Department of General and Pancreatic Surgery, Pancreas Institute, University of Verona Hospital Trust, Verona, Italy; 7 Division of Surgery, Department of Clinical Science, Intervention and Technology, Karolinska Institutet at Karolinska University Hospital, Stockholm, Sweden; 8 Department of Surgery, Newcastle Upon Tyne Hospitals NHS Foundation Trust, Newcastle, UK; 9 Department of Surgery in Linköping and Department of Biomedical and Clinical Sciences, Linköping University, Linköping, Sweden; 10 Department of Academic Surgery, The Royal Marsden Hospital, London, UK; 11 Department of Hepatobiliary and Pancreatic Surgery, Oxford University Hospitals NHS Foundation Trust, Oxford, UK; 12 Faculty of Medicine, University of Birmingham, Birmingham, UK; 13 Division of Oncologic and Mini-Invasive General Surgery, ASST Grande Ospedale Metropolitano Niguarda, Milan, Italy; 14 Department of Surgery, Hospital de Sant Pau, Barcelona, Spain; 15 Department of Surgery, Hellenic Anticancer Hospital “Saint Savvas”, Athens, Greece; 16 Department of Surgery, Miguel Servet University Hospital, Zaragoza, Spain; 17 Hepatobiliary and Pancreatic Surgical Unit, Nouvel Hôpital Civil (NHC), Strasbourg, France; 18 Department of Surgery, Catharina Hospital Eindhoven, Eindhoven, The Netherlands; 19 Department of Surgery, Morriston Hospital, Swansea, UK; 20 Department of Surgery, Hospital del Mar, Barcelona, Spain; 21 Department of Digestive, Oncologic and Metabolic Surgery, Institut Mutualiste Montsouris, Paris, France; 22 Department of Surgery, Martin-Luther University Halle-Wittenberg, Halle (Saale), Germany; 23 Department of Surgery, Pisa University Hospital, Pisa, Italy; 24 Department of Surgery, University Hospital Virgen de la Victoria, Malaga, Spain; 25 Department of Hepatopancreatobiliary and Transplant Surgery, Singapore General Hospital, Duke-National University of Singapore, Singapore, Singapore; 26 Department of Medical Oncology, Amsterdam UMC, University of Amsterdam, Amsterdam, The Netherlands; 27 Oncology Unit, Fondazione IRCCS Ca’ Granda Ospedale Maggiore Policlinico, Milan, Italy; 28 Department of Medical Oncology, Fondazione Poliambulanza, Brescia, Italy; 29 Department of Pathology, University of Oslo, Oslo University Hospital, Oslo, Norway; 30 Department of Pathology, Koç University Hospital and Koç University Research Center for Translational Medicine (KUTTAM), Istanbul, Turkey; 31 Department of Pathology, Fondazione Poliambulanza, Brescia, Italy

## Abstract

**Background:**

Standard lymphadenectomy for pancreatoduodenectomy is defined for pancreatic ductal adenocarcinoma and adopted for patients with non-pancreatic periampullary cancer (NPPC), ampullary adenocarcinoma (AAC), distal cholangiocarcinoma (dCCA), or duodenal adenocarcinoma (DAC). This study aimed to compare the patterns of lymph node metastases among the different NPPCs in a large series and in a systematic review to guide the discussion on surgical lymphadenectomy and pathology assessment.

**Methods:**

This retrospective cohort study included patients after pancreatoduodenectomy for NPPC with at least one lymph node metastasis (2010–2021) from 24 centers in nine countries. The primary outcome was identification of lymph node stations affected in case of a lymph node metastasis per NPPC. A separate systematic review included studies on lymph node metastases patterns of AAC, dCCA, and DAC.

**Results:**

The study included 2367 patients, of whom 1535 had AAC, 616 had dCCA, and 216 had DAC. More patients with pancreatobiliary type AAC had one or more lymph node metastasis (67.2% vs 44.8%; *P* < 0.001) compared with intestinal-type, but no differences in metastasis pattern were observed. Stations 13 and 17 were most frequently involved (95%, 94%, and 90%). Whereas dCCA metastasized more frequently to station 12 (13.0% vs 6.4% and 7.0%, *P* = 0.005), DAC metastasized more frequently to stations 6 (5.0% vs 0% and 2.7%; *P* < 0.001) and 14 (17.0% vs 8.4% and 11.7%, *P* = 0.015).

**Conclusion:**

This study is the first to comprehensively demonstrate the differences and similarities in lymph node metastases spread among NPPCs, to identify the existing research gaps, and to underscore the importance of standardized lymphadenectomy and pathologic assessment for AAC, dCCA, and DAC.

**Supplementary Information:**

The online version contains supplementary material available at 10.1245/s10434-024-15213-z.

Non-pancreatic periampullary cancers (NPPCs) are a group of tumors with a close anatomic relation including ampullary carcinoma (AAC, consisting of the intestinal, pancreatobiliary, and mixed/hybrid subtypes),^1^ distal cholangiocarcinoma (dCCA), and duodenal adenocarcinoma (DAC).^2^ These tumors are relatively rare, but they have a poor prognosis, with reported 5-year survival rates ranging from 30 to 70% for AAC,^3–5^ 18 to 40% for dCCA,^6–9^ and 46 to 71% for DAC.^10–12^ A critical determinant of a worse prognosis for NPPC, influencingthe decision for adjuvant treatment, is lymph node metastases.^10,13,14^ Therefore, adequate lymphadenectomy and pathologic assessment of the resected lymph nodes is crucial for accurate staging and has an impact on postoperative treatment methods such as chemotherapy.

Standard lymphadenectomy, as described by the International Study Group of Pancreatic Surgery (ISGPS), defines the lymph node stations that require resection during pancreatoduodenectomy.^15^ Lymphadenectomy has been standardized for patients with resectable pancreatic ducal adenocarcinoma (PDAC) and was subsequently adopted for patients with NPPC. However, lymphatic drainage of the NPPCs is not necessarily similar to that of PDAC because the embryologic origin of the ventral (with a biliary system) and dorsal pancreas evolve from two different outpouchings of the endodermal lining of the duodenum.^16^ Furthermore, studies that assessed the lymph node metastases patterns for different NPPCs are limited to observational studies with a low number of patients focused on determining the overall lymph node yield rather than specifying which lymph node stations should be included in the lymphadenectomy and identified during pathologic assessment of the resection specimen.

To date, the lymph node metastases patterns of the different NPPCs have not been well characterized or compared with each other. Therefore, this study aimed to evaluate the differences in lymph node metastases patterns among the different NPPCs in order to guide the clinical practice for the surgeon’s lymphadenectomy and pathology examination.

## Methods

### Study Design

This study was a multicenter international retrospective observational cohort study using the database of the international study group on non-pancreatic periampullary cancer (ISGACA; www.isgaca.com) combined with a systematic literature review. The Ethical Committee Brescia approved the study (NP 5269–STUDIO NPPC 15.03.2022). This retrospective study followed the Strengthening the Reporting of Observational Studies in Epidemiology (STROBE) guidelines and checklist.^17^

### Data Collection

Data from the ISGACA database were retrospectively collected from 24 centers located in the United Kingdom (*n* = 7), Italy (*n* = 4), Spain (*n* = 4), Sweden (*n* = 2), the Netherlands (*n* = 2), France (*n* = 2), Germany (*n* = 1), Greece (*n* = 1), and Singapore (*n* = 1).

### Patient Inclusion

The patients included in this study met the following criteria: confirmed pathology report indicating AAC, dCCA, or DAC and underwent curative-intent resection via an open, laparoscopic, robotic or hybrid classic Whipple or pylorus-preserving pancreatoduodenectomy between 2010 and 2021. Cases that involved pancreas-preserving duodenectomy or limited duodenal resection were not considered for inclusion. Patients who had distant metastasis or received neoadjuvant chemotherapy were excluded from analyses. As the study was conducted across multiple centers, local surgical and postoperative clinical protocols were followed.

### Cohorts

The cohorts included in this study were patients with AAC, dCCA, or DAC. The definition of the AAC cohort was based on a subgroup assessment before the main assessment of the study. Within the AAC cohort, two subgroups were compared before the main analyses, including intestinal-type and pancreatobiliary-type AAC, in order to assess whether AAC should be assessed according to subtype or as AAC collectively. Due to the lack of a precise classification for the mixed/hybrid subtype of ampullary carcinoma, this subgroup was not considered helpful in the assessment of the other subgroups. In case no significant differences were found between the intestinal and pancreatobiliary subtypes, the ampullary subgroups were combined including patients with all subtypes (intestinal, pancreatobiliary, mixed/hybrid, or unknown subtype) of AAC. After subgroup analyses of the AAC cohort, the AAC was compared with the dCCA and DAC cohorts.

### Data Definitions

The collected demographic data were sex, age (years), American Society of Anesthesiologists (ASA) classification,^18^ body mass index (BMI [kg/m^2^]), and administration of adjuvant chemotherapy. Resected specimens were evaluated by local certified pathologists, and results were reported per local protocol.^19^ The pathology data were obtained from the local postoperative pathology reports. The techniques used for subtyping were based on histomorphology and supported in some cases by immunohistochemistry.

The definitions of AAC, DAC, dCCA, and PDAC were based on WHO classification,^20^ and pathology examination followed local protocols. Tumor classification followed the seventh edition of the American Joint Committee on Cancer (AJCC) classification.^21^ An R1 resection margin was defined as smaller than 1 mm according to the definition of the Royal College of Pathologists.^19^ Tumor size was measured in millimeters in the postoperative specimen assessment.

The presence of lymph node metastasis, perineural invasion, and lymphovascular invasion and the grade of differentiation were collected. The lymph node ratio was calculated by dividing the total number of metastatic lymph nodes by the total number of lymph nodes harvested during surgery (lymph node yield). In case of lymph node metastasis, the location or locations of the metastases were reported, including the following lymph node stations regardless of the number of lymph node metastases per station: peripancreatic (nos. 13 and 17), infra pyloric (no. 6), common hepatic artery (no. 8), celiac trunk (no. 9), hepatoduodenal ligament (no. 12) and superior mesenteric artery (no. 14),.

### Outcomes

The primary outcome of the study was the percentage of patients with lymph node metastasis in the aforementioned relevant lymph node stations. The reported percentage of lymph node metastasis was calculated by dividing the number of patients with a lymph node metastasis in the concerning lymph node station by the total number of patients who had an N1 (≥1 lymph node metastases) in the same cohort.

### Systematic Literature Review

All the included studies (case series, case-control studies, retrospective cohorts, prospective cohorts, and randomized controlled trials) on lymph node metastasis locations of AAC, dCCA, DAC were identified using Pubmed, Embase (via Ovid), and Cochrane databases. The keywords “ampulla,” “distal bile duct,” “duodenum,” “cancer,” and “lymph node metastases” with all potential synonyms were used in combination to identify all relevant studies until February 2023 (elaboration of the search strategy is available in the supplementary material).

The search was extended with a manual evaluation of relevant references used in the included articles. The results from the included studies, the occurrence of lymph node metastases in the different lymph node stations, were collected and summarized. The studies were separated per NPPC subtype, and the presence or absence of metastases was assessed in each lymph node station available in the study. Binary data (lymph node metastasis or no lymph node metastasis) were collected for each lymph node station, and the prevalence and distribution of lymph node metastases were determined.

### Statistical Analyses

The data collected for this study were analyzed using R (version 4.2.3 for macOS). Missing data were excluded from analyses. The statistical significance level was set at a *P* value lower than 0.05 for the reporting of all results. Normally distributed data are presented as means with standard deviations, whereas non-normally distributed data are presented as medians with interquartile ranges (IQRs). Categorical variables are reported as frequencies and proportions and compared using the chi square-test or Fisher’s test as appropriate. Numeric data were compared using Student’s *t* test for normally distributed data and the Mann-Whitney *U* test for non-normally distributed data.

The individual effect of the lymph node metastases per lymph node station on overall survival was assessed for all the all patients with one or more lymph node metastases using uni- and multivariate Cox hazard models. All lymph node station variables were entered into the Cox proportional hazard regression model, and the variable for the different tumor subgroups was added as an individual covariate. Univariate Cox hazard model (enter method) analysis was performed, and subsequently, all variables with a *P* value lower than 0.20 were selected for multivariate analyses. Coefficients, hazard ratios (HRs), standard errors, *z* values, and *P* values for each variable of the final model are presented.

## Results

### Literature Review

The systematic literature search strategy and study selection can be found in the supplementary materials. After screening for eligibility, 13 studies were included (8 for AAC,^22–29^ 4 for dCCA,^26,30–32^ and 3 for DAC).^26,33,34^ Hempel et al.^26^ assessed all three NPPCs (Table 2). In the included studies, the total numbers of AAC, dCCA, and DAC patients presenting with one or more lymph node metastases were respectively 476, 241, and 78. None of the studies compared the lymph node metastases patterns among the NPPCs, and none of the studies assessed lymph node spread among the ampullary subtypes. The percentages varied widely due to the use of different methods in data collection and reporting of the outcomes. In Table 2, an overview of the current literature and lymph node metastasis distribution is displayed.

### Cohort Study

#### Baseline Characteristics

Patients for this study were included from 24 centers across nine countries, resulting in a total of 2367 patients (Table 1). Of these patients, 1535 had AAC (including 480 with the intestinal subtype and 568 with the pancreatobiliary subtype), 626 had dCCA, and 216 had DAC (Table 1). One or more lymph node metastases were found in 871 (59.9%) of the patients with AAC, 298 (61.0%) of the patients with dCCA, and 121 (65.0%) of the patients with DAC.

**Table 1:** Baseline characteristics of 2367 patients with a non-pancreatic periampullary cancer and at least one lymph node metastasis

Total 2367	AmpIT *n* (%)	AmpPB *n* (%)	*P* Value	Ampulla Ca. *n* (%)	dCCA *n* (%)	DAC *n* (%)	*P* Value
*n*	480	568		1535	616	216	
Female sex	209 (43.5)	231 (40.7)	0.360	646 (42.2)	219 (35.6)	87 (40.3)	0.017
ASA (½)	295 (67.2)	348 (70.6)	0.376	882 (69.6)	404 (69.0)	152 (70.3)	0.963
ASA (¾)	144 (32.8)	145 (29.4)		385 (30.4)	180 (31.0)	64 (29.6)	
Median age: years (IQR)	68 (61–74)	68 (61–74)	0.776	68 (61–74)	68 (61–74)	67 (60–73)	0.478
Median BMI: kg/m^2^ (IQR)	25.50 (23.00–28.07)	25.00 (22.80–28.03)	0.212	25.13 (22.90–28.09)	24.80 (22.30–27.72)	25.00 (23.00–27.73)	0.058
T1/2	289 (62.5)	177 (32.0)	< 0.001	661 (45.5)	129 (26.3)	27 (14.5)	< 0.001
T3/4	175 (37.5)	376 (68.0)		793 (54.5)	360 (73.7)	159 (85.5)	
N0 stage	256 (55.2)	181 (32.7)	< 0.001	583 (40.1)	191 (39.1)	65 (34.9)	< 0.001
N1/2 stage	208 (44.8)	372 (67.2)		871 (59.9)	298 (61.0)	121 (65.0)	
Median tumor size: mm (IQR)	22 (15–30)	20 (15–30)	0.061	20 (15–30)	20 (15–27)	35 (25–45)	< 0.001
Median positive LNs: *n* (IQR)	0 (0–2)	2 (0–4)	< 0.001	1 (0–3)	1 (0–3)	2 (0–4)	0.044
Median total resected LNs: *n* (IQR)	18 (13–26)	18 (13–26)	0.657	18 (13–26)	19 (13–27)	19 (13–28)	0.175
Lymphovascular invasion	205 (43.2)	342 (60.7)	< 0.001	804 (53.8)	326 (57.4)	111 (52.6)	0.287
Perineural invasion)	117 (24.7)	295 (52.4)	< 0.001	607 (40.6)	451 (78.6)	98 (47.8)	< 0.001
R0 resection margin	435 (91.6)	466 (82.3)	< 0.001	1314 (86.2)	433 (71.0)	189 (88.3)	< 0.001

AmpIT, ampullary adenocarcinoma intestinal type; AmpPB, ampullary adenocarcinoma pancreatobiliary type; Ampulla Ca., ampullary carcinoma; dCCA, distal cholangiocarcinoma; DAC, duodenal adenocarcinoma; ASA, American Society of Anesthesiologists; IQR, interquartile range; BMI, body mass index; T stage and N stage, tumor stage following the AJCC 7th classification system; LN, lymph node; R0, >1-mm margin

#### Subtypes of AAC

The lymph node metastases patterns of the intestinal and pancreatobiliary subtypes of AAC were compared before the primary outcomes and are reported in Table 2. Although the pancreatobiliary-type AAC had more lymph node metastases (372, 67.2%) than the intestinal type (*n* = 208 [44.8%]; *P* <0.001; Table 1), a comparable lymph node metastasis pattern was found between the intestinal and pancreatobiliary types of ampullary cancer (*P* > 0.05; Table 2). Therefore, both subtypes were combined into a collective cohort for further analyses.

**Table 2:** Systematic literature review and retrospective cohort assessment of lymph node metastasis patterns among non-pancreatic periampullary cancers (NPPCs)

LNM for NPPC	Total n	N1-2 pte	13/17 Peripanc.		13 - post.panc.duod		17 - ant.panc.duo		6 Infrapyl	
			*n*	%	*n*	%	*n*	%	*n*	%
*Ampullar/ studies*										
Shirai 1997	39	21			20	95%	3			
Yoshida [23]	35	20			20	100%				
Kayahara [24]	51	23			10	43%	2	9%	0	0%
Lee [25]	52	32			26	81%	11	34%		
Hempel [26]		25								
Matsui [27]	114	48	48	100%						
Zhang [28]	110	84			68	81%	66	79%		
Takagi [29]	75	14	10	71%					0	0%
*Total*	*476*	*267*	*58*	***94%***	*144*	***80%***	*82*	***59%***	*0*	***0%***
*Distal cholangio studies*										
Yoshida [30]	42	25			25	100%				
Kato [31]	94	40			26	65%	3	8%	1	3%
Hempel [32]		35								
Kurahara [33]	105	46			33	72%	9	20%		
*Total*	*241*	*146*			*84*	***76%***	*12*	***14%***	*1*	***3%***
*Duodenal studies*										
Sakamoto [34]	51	26	12	46%					4	15%
Hempel [35]		5								
Nishio [36]	27	11							3	27%
*Total*	*78*	*42*	*12*	***46%***					*7*	***19%***
*ISGACA database*										
Ampullary (all subtypes)	–	740	700	94.7%					0"	0%
Ampullary Intestinal	–	179	171	95.5%					0	0%
Ampullary pancreatobiliary	–	335	318	95.2%					0	0%
P - value (AmpIT vs AmpPB)	–		0.870						n.a.	
Distal cholangiocarcinoma	–	227	210	94.2%					6	2.7%
Duodenal adenocarcinoma	–	101	90	90.0%					5	5.0%
P - value (Amp vs dCCA vs DAC)			0.167						**<0.001**	

LNM for NPPC	Total n	N1-2 pte	8 - com.hep.art		9 - Celiac trunk		12 - hepatoduo.lig		14 - sup.mesent.art		16 -para-aortic	
			*n*	%	*n*	%	*n*	%	*n*	%	*n*	%
*Ampullar/ studies*												
Shirai 1997	39	21	3	14%					3	15%	3	60%
Yoshida [23]	35	20										
Kayahara [24]	51	23	1	4%	0	0%	0	0%	9	39%	0	0%
Lee [25]	52	32	0	0%			0	0%	2	6%	1	4%
Hempel [26]		25									2	8%
Matsui [27]	114	48					2	4%	4	8%	5	10%
Zhang [28]	110	84	48	57%	11	13%	58	69%	7	8%	2	2%
Takagi [29]	75	14	0	0%			1	7%	4	29%	2	14%
*Total*	*476*	*267*	*52*	***30%***	*11*	**10%**	*61*	***30%***	*29*	***13%***	*15*	***7%***
*Distal cholangio studies*												
Yoshida [30]	42	25					25					
Kato [31]	94	40	7	18%	1	3%	17	43%	6	15%	1	3%
Hempel [32]		35									5	14%
Kurahara [33]	105	46	5	11%			19	41%	1	2%	2	4%
*Total*	*241*	*146*	*12*	***14%***	*1*	**3%**	*61*	***71%***	*7*	***8%***	*8*	***7%***
Duodenal studies												
Sakamoto [34]	51	26					0	0%	7	27%	4	15%
Hempel [35]		5									0	0%
Nishio [36]	27	11					5	45%	2	18%		
*Total*	*78*	*42*					*5*	***14%***	*9*	***24%***	*4*	***13%***
*ISGACA database*												
Ampullary (all subtypes)	–	740	35	4.7%	12	1.6%	47	6.4%	62	8.4%		
Ampullary Intestinal	–	179	9	5,0%	3	1.7%	9	1.7%	8	4.5%		
Ampullary pancreatobiliary	–	335	18	5.4%	8	2.4%	16	4.8%	16	4.8%		
P - value (AmpIT vs AmpPB)	–		0.867		0.595		0.899		0.875			
Distal cholangiocarcinoma	–	227	15	6.7%	4	1.8%	29	**13.0%**	26	**11.7%**		
Duodenal adenocarcinoma	–	101	9	9.0%	4	4.0%	7	7.0%	17	**17.0%**		
P - value (Amp vs dCCA vs DAC)			0.149		0.258		**0.005**		**0.015**			

*Upper part.*: systematic literature review for lymph node metastasis pattern for ampullary cancer (AAC)^22–29^, distal cholangiocarcinoma (dCCA),^26,30–32^ and duodenal adenocarcinoma (DAC),^26,33,34^. *Lower part*: Location of the lymph node metastases in patients with at least one lymph node metastasis.

LNM, lymph node metastases; Peripanc., peripancreatic; ant.panc.duo, anterior pancreatoduodenal ligament; infrapyl, infrapyloric; com.hep.art, common hepatic artery; hepatoduo.lig, hepatoduodenal ligament; sup.mesent.art, superior meseteric artery; pte, no. of patients; Amp, ampullary adenocarcinoma; dCCA, distal cholangiocarcinoma; DAC, duodenal adenocarcinoma.

#### Comparison of the NPPCs

**Fig. 1:**
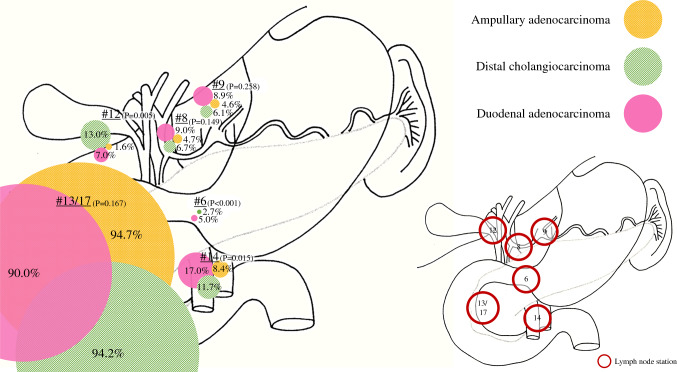
Distribution of lymph node metastases among ampullary adenocarcinoma, distal cholangiocarcinoma, and duodenal adenocarcinoma. Lymph node stations: peripancreatic (nos. 13 and 17), infra pyloric (no. 6), common hepatic artery (no. 8), celiac trunk (no. 9), hepatoduodenal ligament (no. 12), superior mesenteric artery (no. 14). Significant differences are found in lymph node stations 6, 12, and 14 (*P* < 0.05). The percentages correspond with those in Table 2

For AAC, dCCA, and DAC, the majority of lymph node metastases were located in peripancreatic lymph node stations 13 (94.7%), 17 (94.2%), or both (90.0%) (*P* = 0.167, Table 2). The infra-pyloric lymph node station 6 was more frequently affected in DAC (5.0%) than in dCCA (2.7%) or AAC (0%) (*P* < 0.001). The hepatoduodenal ligament station 12 was more frequently affected in dCCA (13.0%) than in DAC (7.0%) or AAC (6.4%) (*P* = 0.005). The superior mesenteric artery station 14 was more frequently affected in DAC (17.0%) than in dCCA (11.7%) or AAC (8.4%) (*P* = 0.015). Figure 1 gives a visual presentation of the differences in lymph node metastases patterns among the different NPPCs.

#### Lymph Node Metastases as Predictors for Overall Survival

Table 3 demonstrates uni- and multivariate Cox regression analyses for each lymph node station associated with overall survival. The variables for tumor type, lymph node station 9, and lymph node station 14 showed a potential influence on survival, with a *P* value lower than 0.20 in univariate analyses, and were included in the multivariate model. The multivariate Cox proportional hazard model showed an individual predictive effect of one or more lymph node metastases on overall survival in lymph node station 14 regardless of tumor type or other lymph node metastases (HR, 1.559; *P* = 0.009).

**Table 3:** Influence of a lymph node metastasis per lymph node station on overall survival using uni- and multivariate Cox proportional hazard model for overall survival

	Univariate					Multivariate				
	Coeff.	HR	SE (coeff.)	*z* Value	*P* Value^a^	Coeff.	HR	SE (coeff.)	*z* Value	*P* Value^a^
AmpIT vs AmpPB	0.5492	1.732	0.1504	3.651	**< 0.001**	0.5526	1.738	0.1508	3.666	**< 0.001**
AmpIT vs dCCA	0.5902	1.804	0.1568	3.765	**< 0.001**	0.5536	1.740	0.1576	3.512	**< 0.001**
AmpIT vs DAC	0.2466	1.280	0.2013	1.225	0.221	0.1555	1.168	0.2046	0.760	0.447
Peripancreatic (station 13 or 17)	0.2488	1.283	0.2053	1.212	0.226					
Infrapyloric (station 6)	0.1575	1.171	0.3809	0.414	0.679					
Common hepatic artery (station 8)	0.0181	1.018	0.1946	0.093	0.926					
Celiac trunc (station 9)	0.4644	1.591	0.2712	1.712	0.087	0.3891	1.476	0.2867	1.357	0.175
Hepatoduodenal ligament (station 12)	0.1362	1.146	0.1535	0.887	0.375					
Superior mesenteric artery (station 14)	0.3336	1.396	0.1409	2.368	**0.018**	0.4437	1.559	0.1694	2.619	**0.009**

Coeff, the beta coefficient; HR, hazard ratio; SE, standard error; AmpIT, intestinal-type ampullary adenocarcinoma; AmpPB, pancreatobiliary-type ampullary adenocarcinoma; dCCA, distal cholangiocarcinoma; DAC, duodenal adenocarcinoma

^a^Bold type indicates statistically significant values (*P* < 0.05).

## Discussion

This international multicenter cohort study showed that NPPC most frequently metastasizes to the peripancreatic lymph node stations. Lymph node station 8 was more frequently affected in patients with dCCA, whereas stations 6 and 14 were more frequently affected in patients with DAC. Lymph node metastases in station 14 were found to be an individual predictor for survival. The rate of lymph node metastases differed between the intestinal and pancreatobiliary types of ampullary cancer, whereas there was no difference in lymph node metastases patterns.

This study combined a large retrospective cohort study with a systematic literature review. The systematic literature review showed the inability of current existing literature to provide a comprehensive comparison of lymph node metastases patterns among NPPCs. Despite the lack of adequate conclusive insights in lymph node metastases patterns among different NPPCs, the literature review provided an overview of the current evidence, showed heterogenic outcomes, and identified the research gap in this topic.

The ISGPS defined the standard lymphadenectomy, which included lymph node stations 5, 6, 8a, 12b1, 12b2, 12c, 13a, 13b, 14a, 14b, 17a, and 17b.^15^ However, the standard lymphadenectomy was based on patients with PDAC. Overall, the differences in lymph node metastases patterns found between AAC, dCCA, and DAC in this study showed the importance of a standardized adequate lymphadenectomy during pancreatoduodenectomy and supported the ISGPS standard lymphadenectomy for AAC, dCCA, and DAC.

The existing literature already suggested that the majority of lymph node metastases are found in peripancreatic lymph nodes stations 13 and 17 for AAC,^22,23,25,27–29^ dCCA,^30–32^ and DAC.^33^ This study confirmed that most lymph node metastases are located in peripancreatic stations 13 and 17 of all NPPCs. However, it is important to note that despite the clear majority of affected lymph nodes in lymph node stations 13 and 17, these do not act as “sentinel nodes.”

Differences in the proportions of affected lymph nodes were found in lymph node stations 6, 12, and 14. Lymph node station 12 was found to have a metastasis more often in case of dCCA, and lymph node stations 6 and 14 were found to have a metastasis more often in case of DAC. Because most pancreatic centers perform profound pathology assessment of surgical specimen, this evidence does not directly demand alternation in current clinical care. However, the observed variation among NPPCs in lymph node metastases patterns should serve as a guide for the pathologist, indicating that the identification of crucial lymph node stations in the specimen differs per NPPC. In cases with no identification of relevant lymph nodes, a revision of the specimen could be considered. Future studies should assess whether more profound lymph node examination (i.e., multiple-section examination or additional staining) of these specific lymph nodes leads to the identification of otherwise missed micro-metastases.

Lymph node metastases in pancreatic cancer and periampullary cancer are associated with reduced survival rates.^10,13,14^ Nevertheless, the clinical relevance of lymph node metastases in each of the lymph node stations in the peripancreatic region requires further evaluation. This study evaluated the individual impact of metastases in each lymph node station on overall survival while accounting for the NPPC tumor type. The analysis indicated that a metastasis in lymph node station 14 significantly and independently predicts overall survival. This predictive value results in extra importance for adequate lymphadenectomy around the superior mesenteric artery and for identification of lymph station 14 in pathology examination. With adequate documentation of resection and assessment, future studies can assess whether resection of lymph node 14 holds a therapeutic value or may contribute in selecting patients who could benefit most from adjuvant chemotherapy.

The intestinal and pancreatobiliary AAC types differ regarding prognosis and response to chemotherapy.^35–37^ However, there was no evidence on the distribution of lymph node metastases between the ampullary subtypes. This study demonstrated that the occurrence of lymph node metastases, high T stage, lymphovascular invasion, and R1 resections was significantly more prevalent in the pancreatobiliary-type than in the intestinal-type AAC, whereas no differences were observed in the pattern of lymph node metastases. This suggests that despite the differences in tumor aggressiveness and behavior between the subtypes,^38^ the lymphatic drainage of the intestinal and pancreatobiliary AAC types appears to be comparable. This finding contributes to a deeper understanding of the tumor biology of AAC subtypes.

For DAC specifically, a potential surgical option is the segment resection. Sakamoto et al.^33^ and Nishio et al.^34^ both show that a segmental resection is only an option for T1(a), very distal duodenal tumors, or patients unfit for pancreatoduodenectomy. The results of this study demonstrated the diverse lymph node metastases pattern of the DAC. Especially in high-grade DAC, it is not recommended to perform a segmental resection over a pancreatoduodenectomy.

An extended lymphadenectomy is not recommended for patients with pancreatic ductal adenocarcinoma (PDAC) because it can result in increased morbidity and worse surgical outcomes without any survival benefits.^15,39^ Some studies claim that because the survival rate is significantly worse for patients with lymph node metastases in station 16, it should be classified as a distant metastases and categorized as M1 in the tumor-node-metastasis (TNM) classification.^40^ The effect of lymph node metastases on lymph node station 16 for AAC, dCCA, and DAC has been only marginally researched.^25,26,29,41^ Hempel et al.^26^ demonstrated that for NPPCs, a lymph node metastasis in station 16 did not limit overall survival in a small single-center setting. Yet, it is common practice not to resect lymph node station 16 for NPPCs, similar to PDAC. Future studies should explore the effect of lymph node metastases in station 16 for AAC, dCCA, and DAC individually and assess whether the resection of station 16 would result in survival benefit and better staging, or whether similar to PDAC, it does not seem to benefit the patients.

The results of this study should be interpreted with some limitations in mind. First, due to the retrospective character of the study, specimens could not be revised, and not all pathologists followed standard reporting on found lymph node locations. Consequently, if lymph nodes were absent or not found in the specimen, they were likely categorized as negative. Despite the assumption that missing lymph nodes are mostly negative due to their small size, a small possibility of overlooked positive nodes remains, potentially leading to an underestimation of the number of positive lymph nodes. However, we maintain the belief that these situations were uncommon, and considering the high numbers involved, they did not have a significant impact on the study outcomes.

Second, station 8 is not separated for anterior and/or posterior in our database. However, this study suggests that mainly for dCCA and DAC, lymph node station 8 plays an important role. It is therefore important to pay attention to resection of both lymph node stations 8a and 8p during pancreatoduodenectomy, in which station 8a is more frequently resected and 8p is mainly collected separately.

Third, the minimal lymph node yield was not addressed in this study. It is commonly mentioned that a minimum of 15 lymph nodes should be resected during the procedure. Although lymph node yield is considered an indicator of surgical quality, the primary focus should be on resecting all important stations rather than on solely aiming for a specific number.

Fourth, the availability of the TNM eighth classification was limited to the recent years. Consequently, the decision was made to use the seventh TNM classification based on data completeness. Table S1 in the supplementary material shows the comparison between the seventh and eighth TNM classifications for AAC, dCCA, and DAC.

Fifth, the mixed or hybrid subtypes are described in the literature as important subtypes of AAC.^1^ However, definitions of these subtypes varied among centers, so it could not be stated with certainty that a tumor categorized as mixed was not in reality an intestinal or pancreatobiliary AAC type. Therefore, this cohort was considered too heterogenic to be included in the AAC subgroup comparison. Because certified pathologists diagnosed all AAC cases, those labeled mixed/hybrid-type AAC were included in the AAC cohort.

Sixth, there are no data on the exact location of the periampullary duodenal adenocarcinoma. Future studies should incorporate this in order to assess the correlation between location and lymph node spread.

Finally, due to the multicenter international approach, local regional differences in perioperative patient care were inevitable.

Despite its limitations, this study is the first, largest, and most comprehensive evaluation of lymph node metastases patterns in all NPPCs to date, including both a systematic literature search and a large number of patients from multiple centers and countries. Due to the combination, this study provides all data currently available on the topic. Furthermore, the sufficient number of patients with all NPPCs allowed reporting on the lymph node metastases patterns per NPPC individually and making of comparisons among AAC, dCCA, and DAC. In addition, the inclusion of subgroups for AAC provided more detailed information on differences in lymph node metastases patterns between intestinal-type and pancreatobiliary-type cancers.

In conclusion, this study demonstrated the distribution of lymph node metastases in AAC, dCCA, and DAC individually, aiding in the understanding of tumor biology. The differences in lymph node metastases found among NPPCs can guide the pathologist in targeted identification of lymph nodes during specimen assessment. As shown for PDAC, this study confirms the value of standardized lymphadenectomy for all periampullary cancers. Moving forward, future research should explore whether surgical techniques can be further personalized and whether targeted lymph node identification decreases otherwise missed micro-metastases.

### Supplementary Information

Below is the link to the electronic supplementary material.

Supplementary file1 (DOCX 71 KB)
