# Supplementary material for: Differences in Lymph Node Metastases Patterns Among Non-pancreatic Periampullary Cancers and Histologic Subtypes: An International Multicenter Retrospective Cohort Study and Systematic Review
Source: Ann Surg Oncol. 2024 Apr 11;31(7):4654–64. doi: 10.1245/s10434-024-15213-z (PMC11164734; doi:10.1245/s10434-024-15213-z)
Supplement: Supplementary file 1 — Supplementary file1 (DOCX 71 KB) [file 10434_2024_15213_MOESM1_ESM.docx]

**The differences in lymph node metastases patterns among non-pancreatic periampullary cancers and histological subtypes:
an international multicenter retrospective cohort study and systematic review**

**Supplementary**

**Systematic literature search**

Search:

Pubmed

General:

[Ampulla] OR [Distal bile duct] OR [duodenum] OR [non-pancreatic periampullary] OR [periampullary]

AND

[Malignant disease]

AND

Lymph node yield, lymph node metastasis, lymph node spread, lymph node spread pattern, number of resected lymph nodes

Search terms

(“Ampulla”[Title/Abstract] OR "ampullary”[Title/Abstract] OR "Ampulla of Vater"[Mesh] OR "distal bile duct”[Title/Abstract] OR "distal cholangio*”[Title/Abstract] OR "duodenal”[Title/Abstract] OR "duodenum”[Title/Abstract] OR “non-ampullary duodenal”[Title/Abstract] OR “non-ampullary periampullary”[Title/Abstract] OR "Duodenum"[Mesh] OR “non-pancreatic periampullary”[Title/Abstract] OR “periampullary”[Title/Abstract] OR “pancreatic cancer”[Title/Abstract]) AND (“Adenocarcinoma”[Title/Abstract] OR “carcinoma”[Title/Abstract] OR “malignant”[Title/Abstract] OR “cancer”[Title/Abstract] OR “tumor”[Title/Abstract] OR “papillary carcinoma”[Title/Abstract] OR “papillary adenocarcinoma”[Title/Abstract] OR “Neoplas*”[Title/Abstract] ) AND

(“Lymph node yield”[Title/Abstract] OR “lymph node metastasis”[Title/Abstract] OR “lymph node spread”[Title/Abstract]

OR “number of resected lymph nodes”[Title/Abstract] OR “Pattern of lymph node”[Title/Abstract] OR “metastasis spread”[Title/Abstract] )

EMBASE

#1

'Ampulla':ab,ti,kw OR 'ampullary':ab,ti,kw OR 'distal bile duct':ab,ti,kw OR 'distal cholangiocarcinoma':ab,ti,kw OR 'duodenal':ab,ti,kw OR 'duodenum':ab,ti,kw OR 'non-ampullary duodenal':ab,ti,kw OR 'non-ampullary periampullary':ab,ti,kw OR 'non-pancreatic periampullary':ab,ti,kw OR 'periampullary':ab,ti,kw

#2

'Adenocarcinoma':ab,ti,kw OR 'carcinoma':ab,ti,kw OR 'malignant':ab,ti,kw OR 'cancer':ab,ti,kw OR 'tumor':ab,ti,kw OR 'papillary carcinoma':ab,ti,kw OR 'papillary adenocarcinoma':ab,ti,kw OR 'Neoplasia':ab,ti,kw

#3

'Lymph node yield':ab,ti,kw OR 'lymph node metastasis':ab,ti,kw OR 'lymph node spread':ab,ti,kw

OR 'number of resected lymph nodes':ab,ti,kw OR 'Pattern of lymph node':ab,ti,kw OR 'metastasis spread':ab,ti,kw

**PRISMA 2020 flow diagram for new systematic reviews which included searches of databases and registers only**

**Identification of studies via databases and registers**

Records removed *before screening*:

Duplicate records removed

(n = 2139)

Records identified from*:

PubMed (n = 1744)

Embase (n = 1200)

Web of Science (1859)

Total (n = 4803)

**Identification**

Records excluded**

(n = 2651)

Records included through other routes (n = 0)

Records screened

(n = 2664)

**Screening**

Studies included in review

(n = 13)

**Included**

*From:* Page MJ, McKenzie JE, Bossuyt PM, Boutron I, Hoffmann TC, Mulrow CD, et al. The PRISMA 2020 statement: an updated guideline for reporting systematic reviews. BMJ 2021;372:n71. doi: 10.1136/bmj.n71

For more information, visit: <http://www.prisma-statement.org/>

**Systematic literature search conclusion:**

The systematic literature review conducted in this study showed the inability to provide a comprehensive comparison of lymph node metastases patterns among NPPCs with current existing literature. The literature review was limited because 1) there was no study that compared AAC, dCCA and/or DAC, 2) different inclusion criteria were used among the studies such as inclusion of all patients with the concerning NPPC, only patients with lymph node metastases or a case-control design, 3) some studies assessed the total number of positive lymph nodes regardless of the number of affected patients while others only assessed the spread of lymph node metastases per patient and 4) not all studies examined all lymph node stations, making it difficult to compare frequencies among studies. The risk of bias assessment resulted in a high risk of bias in all studies due to the retrospective design, non-standardized pathology assessment and low number of included patients.

**Table S1:** Elaborate differentiation between T and N stage of the 7^th^ and 8^th^ TNM classification

|  | **Ampulla AC** | **distal cholangioca.** | **Duodenal AC** | **P - value** |
| --- | --- | --- | --- | --- |
| *2367* | 1535 | 616 | 216 |  |
| *T stage 7th TNM, n (%)* |  |  |  | <0.001 |
| *1* | 185 (12.7) | 33 (6.7) | 10 (5.4) |  |
| *2* | 476 (32.7) | 96 (19.6) | 17 (9.1) |  |
| *3* | 463 (31.8) | 351 (71.8) | 75 (40.3) |  |
| *4* | 330 (22.7) | 9 (1.8) | 84 (45.2) |  |
| *N stage 7th TNM, n (%)* |  |  |  | <0.001 |
| *0* | 583 (40.1) | 191 (39.1) | 65 (34.9) |  |
| *1* | 871 (59.9) | 298 (61.0) | 83 (44.6) |  |
| *2* |  |  | 38 (20.4) |  |
| *T stage 8th TNM, n (%)* |  |  |  | <0.001 |
| *1a* | 91 (10.2) | 62 (13.7) | 9 (4.4) |  |
| *1b* | 98 (11.0) |  | 6 (2.9) |  |
| *2* | 255 (28.6) | 135 (25.4) | 24 (11.8) |  |
| *3a* | 214 (24.0) | 328 (61.9) | 103 (50.5) |  |
| *3b* | 182 (20.4) |  |  |  |
| *4* | 52 (5.8) | 6 (1.1) | 62 (30.4) |  |
| *N stage 8th TNM, n (%)* |  |  |  | <0.001 |
| *0* | 376 (41.9) | 225 (40.0) | 74 (34.9) |  |
| *1* | 395 (44.0) | 245 (43.6) | 72 (34.0) |  |
| *2* | 127 (14.1) | 92 (16.4) | 66 (31.1) |  |

Abbreviations: DAC, Duodenal adenocarcinoma; Ampulla IT, Ampullary adenocarcinoma intestinal-type; Ampulla PB, Ampullary adenocarcinoma pancreatobiliary-type; Ampulla Ca., ampullary carcinoma; Distal cholangnioca., distal cholangiocarcinoma; Duodenal AC, duodenal adenocarcinoma; T-stage, N-stage and Tumor stage following the AJCC 7th classification system
